# Spatial inequalities in skilled birth attendance in India: a spatial-regional model approach

**DOI:** 10.1186/s12889-021-12436-7

**Published:** 2022-01-12

**Authors:** Prem Shankar Mishra, Debashree Sinha, Pradeep Kumar, Shobhit Srivastava

**Affiliations:** 1 Research Scholar, Population Research Centre, Institute for Social and Economic Change, Bengaluru, Karnataka 560072 India; 2 Research Scholar, Department of Development Studies, International Institute for Population Sciences, Mumbai, Maharashtra 400088 India; 3 Research Scholar, Department of Survey Research & Data Analytics, International Institute for Population Sciences, Mumbai, Maharashtra 400088 India

**Keywords:** Skilled birth attendant, Inequality, Spatial analysis, LISA, BiLISA, India

## Abstract

**Background:**

Despite a significant increase in the skilled birth assisted (SBA) deliveries in India, there are huge gaps in availing maternity care services across social gradients - particularly across states and regions. Therefore, this study applies the spatial-regression model to examine the spatial distribution of SBA across districts of India. Furthermore, the study tries to understand the spatially associated population characteristics that influence the low coverage of SBA across districts of India and its regions.

**Methods:**

The study used national representative cross-sectional survey data obtained from the fourth round of National Family Health Survey, conducted in 2015-16. The effective sample size was 259,469 for the analysis. Moran’s I statistics and bivariate Local Indicator for Spatial Association maps were used to understand spatial dependence and clustering of deliveries conducted by SBA coverage in districts of India. Ordinary least square, spatial lag and spatial error models were used to examine the correlates of deliveries conducted by SBA.

**Results:**

Moran’s I value for SBA among women was 0.54, which represents a high spatial auto-correlation of deliveries conducted by SBA over 640 districts of India. There were 145 hotspots for deliveries conducted by SBA among women in India, which includes almost the entire southern part of India. The spatial error model revealed that with a 10% increase in exposure to mass media in a particular district, the deliveries conducted by SBA increased significantly by 2.5%. Interestingly, also with the 10% increase in the four or more antenatal care (ANC) in a particular district, the deliveries conducted by SBA increased significantly by 2.5%. Again, if there was a 10% increase of women with first birth order in a particular district, then the deliveries conducted by SBA significantly increased by 6.1%. If the district experienced an increase of 10% household as female-headed, then the deliveries conducted by SBA significantly increased by 1.4%.

**Conclusion:**

The present study highlights the important role of ANC visits, mass media exposure, education, female household headship that augment the use of an SBA for delivery. Attention should be given in promoting regular ANC visits and strengthening women’s education.

**Supplementary Information:**

The online version contains supplementary material available at 10.1186/s12889-021-12436-7.

## Background

Worldwide in 2015, an estimated 30,3000 women died from pregnancy-related causes [1]. Although the Sustainable Development Goals (SDGs) targets to reduce the global maternal mortality ratio to less than 70 per 1,00,000 live births by 2030, countries that belong particularly to WHO’s (World Health Organization) regions such as the Sub-Saharan-Africa region and South Asia region will be experiencing a high maternal mortality ratio (MMR) [2, 3]. Within the South Asian countries (region), India alone contributes to the highest MMR [3]. Although the MMR has declined from 130 in 2014-2016 to 122 in 2015-17 in India, however, this decline has been observed to be most significant in the Empowered Action Group (EAGs) states and Assam (also known as low performing states) from 188 to 175 per 1,00,000 live births. Among the Southern states (known as high performing states), the decline has been from 77 to 72 [4]. This decline is highly linked with increased use of essential health care and quality of care services such as ante-natal care, institutional delivery and skilled birth attendants (SBA). Earlier studies have clearly shown that unskilled birth attendance and delivery at home are associated with high infant and maternal mortality and morbidity [5–8].

Skilled birth attendance (SBA) is important to save the lives of pregnant women and newborns. An SBA is defined as “an accredited health professional such as a midwife, doctor or nurse, who has been educated and trained to expertise in the skills needed to manage normal (uncomplicated) pregnancies, childbirth and the immediate postnatal period, and in the identification, management and referral of complications in women and newborns” [2]. Skilled birth assistance during childbirth can influence the birth outcome and the health of the mother and the newborn [5]. An SBA can manage complications of pregnancy and delivery or refer the mother and/or the baby to the next level of care [5].

Pregnancy-related deaths among women in India still remains unacceptably high [4]. Though substantial progress has been made, however, the inequality in MMR remains wide and persistent across regions and socio-economic groups [3, 9, 10]. The low coverage of the essentials maternity care such as antenatal care (ANC), SBA, and postnatal care (PNC) have a huge impact on mother and newborn survival and also put them at high risk [2, 11]. Delivery by skilled birth attendants (SBAs) at childbirth can significantly reduce the risk of maternal and neonatal deaths attributed to prematurity, intrapartum or postpartum complications [8, 12, 13]. Increasing institutional deliveries is an important factor in reducing maternal and neonatal mortality [14, 15]. Even though the majority of births are attended by doctors (56%), followed by ANMs (Auxiliary Nurse Midwives), nurses, midwives, lady health visitors (LHVs) (25%), and *dais* (Traditional Birth Attendances) (11%), nearly 7% of childbirths are attended by relatives or friends [10]. Further, it varies across districts and regions of India.

Worldwide, the percentage of births attended by skilled personnel is above 90% in three out of the six WHO regions. However, in certain regions that are lacking in skilled birth attendance (SBA), an increase in coverage is required, such as in Africa, where the figure remains less than 50% followed by South Asia [2]. In the case of India, SBAs have substantially increased; for example, between 1992 and 93 and 2005-06, skilled birth assisted deliveries increased by 13 percentage points (from 36 to 49%), and subsequently, it increased by 81% in 2015-16 [10, 16]. Nevertheless, understanding the regional disparities in skilled birth attendance is needed as India shows that there are huge gaps in availing maternity care services across social gradients in India and particularly across states and regions [12, 17–20]. There are rural-urban differences in using SBA for deliveries, i.e. 90 and 78%, respectively [10]. Further, socio-economic determinants always play a significant role in widening the gaps in the delivery by SBA among women in India. For instance, while only 64% of women in the lowest wealth quintile had an SBA for delivery, 96% of women in the highest wealth quintile availed of the service. Birth preparedness and woman’s age at delivery are also associated with SBA [21–24]. Similar patterns can also be seen across social groups and mother’s levels of education [10]. Furthermore, the inequalities that emerged in availing SBA within the state and between states or regions are highly pervasive in India.

Accessing universal maternity care in India depends on multiple factors. These can be at the individual level or family or community levels. At the community level, the shortage of community skilled health workers, such as doctors, nurse/midwives, and other professional staff and the unavailability of physical infrastructures at the facility levels along with poor attitude and unethical behavior among health workers could further influence the quality of care where pregnant women do not seek health care [13, 14, 25].

Although the public healthcare systems in India have significantly contributed towards an increase in the coverage of institutional delivery care and SBA [20], however, there is a significant lag in the equality and equity part that still persist across regions and socio-economic groups [7, 26–28]. Therefore, to achieve faster and more equitable maternity care services and improve the maternal and child health outcomes across the country and regions, the Government of India launched the National Rural Health Mission (NRHM) in 2005, which later got expanded into the National Health Mission (NHM) in 2013 [9, 20]. The program aimed to enhance and strengthen the infrastructural and human resources across public health facilities to expand coverage of maternal and child health care services in the country. Evidence shows that regional inequality in the distribution of health infrastructure and human resources for health has created a low quality of care for them [29–32]. Besides, the key constraints in providing comprehensive emergency obstetric care services to pregnant women residing in rural and remote areas are the non-availability of obstetricians, gynecologists, skilled midwives [29, 32, 33]. Furthermore, geographical factors have always played a significant role in accessing equitable quality maternity care services not only in India but also in other developing countries [11, 13, 15, 34].

Few studies in the Indian context have examined determinants related to governance and government policies in maternal and child healthcare studies, which are often measured at higher administrative levels, but it has failed to focus at regional and district levels [7, 31, 35]. Socio-economic and demographic factors are closely associated with quality and essential maternity care utilization. Furthermore, demographic variables such as a woman’s age, parity and birth order may also influence her in the utilization of an SBA across communities or regions [23, 36, 37]. Again, studies have often ignored to understand the role of factors at the community and district-level in utilizing the SBA in the country [7, 26, 38, 39]. There are also factors that are associated with inaccessibility and under-utilization of SBA across different regions, districts, and, more specifically, between high performing and low performing states in India. These factors are further associated with geographical factors.

To better understand the distribution of SBA in India, therefore, a spatial-regional approach is required. Furthermore, to identify the determining factors in India as a whole and particularly across spatial-regional context, the study uses a spatial regression model to explain how the equity-relative influencing determinants operate in the unequal distribution of SBA.

## Methods

The study used a nationally representative cross-sectional survey data obtained from the fourth round of National Family Health Survey (NFHS-4) conducted in 2015-16 [10]. The duration for data collection was from 20 January 2015 to 4 December 2016, and the respondents’ information was collected for five years prior to the survey [10]. The survey gathered information on maternal and child health that includes socio-demographic characteristics of the child and reproductive characteristics of female respondents [10]. The survey was carried out across India, covering all 29 states and seven union territories, and yielded estimates for 640 districts for the first time. NFHS-4 adopted a two-stage stratified sampling design for the selection of the sample [10]. In all, 28,586 Primary Sampling Units (PSUs) were selected across the country in NFHS-4, of which fieldwork was completed in 28,522 clusters (Data collection was conducted in two phases from 20 January 2015 to 4 December 2016). The primary sampling units (PSUs) were chosen using the 2011 census as the sampling frame [10]. In rural areas, PSUs were villages and in urban areas, Census Enumeration Blocks (CEBs). PSUs with fewer than 40 households were connected to the PSU closest to them. Villages were chosen from the sampling frame inside each rural stratum with a probability proportional to size (PPS). In each stratum, six approximately equal substrata were created [10]. By combining three substrata based on the projected number of households in each village with two substrata based on the percentage of the population belonging to scheduled castes and scheduled tribes (SCs/STs). PSUs were categorized according to the literacy rate of women aged 6 and up within each specific sampling stratum [10]. Probability Proportional to Size (PPS) sampling was used to select the final sample of PSUs. CEB data was gathered from the Office of the Registrar General and Census Commissioner in New Delhi in urban areas. Selected PSUs with an expected population of at least 300 families were divided into 100-150 household parts [10]. For the survey, two segments were chosen at random using systematic sampling with probability proportional to segment size. As a result, an NFHS-4 cluster is either a PSU or a PSU segment. In the second stage, 22 homes were randomly picked with systematic sampling from each specified rural and urban cluster. The report contains more information on the data source [10]. The survey gathered information from a total of 601,509 households with a response rate of 98%. A total of 699,686 women aged 15-49 years and 112,122 men aged 15-54 years with a response of 97% and 92%, respectively, were interviewed. The present study used the kid’s data file for the analysis (*n* = 259,469).

### Outcome variable

The outcome variable for the present study was delivery assisted by a skilled birth attendant [40]. This was derived from the question ‘who assisted with the delivery?’ Those who had help from a doctor, nurse/midwife, or community health officer/nurse had their responses labelled as assisted skillful delivery. The outcome variable was coded as 1 ‘Yes’ and 0 ‘No’ since it was a dichotomous variable [41].

### Exposure variables

The predictors for this study were undertaken taken after an extensive literature review. It includes educational level (no schooling and educated), women’s exposure to mass media (listened to the radio, how often they read newspapers, and watched television; responses to the questions were: almost every day, at least once a week, less than once a week, or not at all; women were considered to have any exposure to mass media if they had exposure to any of these sources and to have no exposure if they responded with ‘not at all’ for all three sources of media) [42], number of antenatal check-ups (ANC) (less than 4 and 4 or more), birth order (first order and two or more), head of the household (HH) (male and female), caste (Scheduled Caste/Scheduled Tribe and non-Scheduled Caste/Scheduled Tribe). The Scheduled Castes are a group of people who are socially separated and financially/economically disadvantaged as a result of their low caste status in Hindu society. The Scheduled Castes (SCs) and Scheduled Tribes (STs) are among India’s most economically disadvantaged groups. The OBC refers to those who have been labelled “educationally, economically, and socially backward.” In the traditional caste order, the OBCs are regarded as the lowest caste. The “other” caste group is thought to have a greater social position. Other factors were religion (Hindu and non-Hindu), and economic status (poor and non-poor). For the purposes of calculating the wealth index, a household wealth index was used as a proxy of economic status. Households were given scores based on the number and types of consumer goods they own, which range from a television to a bicycle or automobile, as well as dwelling qualities such as drinking water, bathroom facilities, and flooring materials. These figures were derived using principal component analysis. The national wealth quintiles were formed by assigning a score to each usual (de jure) household member, ranking each person in the home population according to their score, and then dividing the distribution into five equal categories, each having 20% of the population [42]; women were considered as poor if they belonged to poorest or poorer wealth quintile and non-poor [43–45], otherwise., Place of residence was coded as urban and rural [10].

### Statistical analysis

The relationship between outcome and exposure variables was investigated using bivariate and multivariate logistic regression analysis. The relationship between the outcome and the independent factors has been established using bivariate analysis. In addition, the model was adjusted for the other factors included in the table in multivariate analysis. AIC (Akaike information criterion) values were used to assess model fit. The model with the lower AIC values is the one that fits best. In addition, univariate and bivariate Moran’s I index measurements, as well as spatial regression models, were used for geographic analysis in terms of assisted skillful delivery among women in India [46]. Moran’s I statistics are used to calculate spatial auto-correlation. It shows how similar or different data points are in relation to their spatial neighbours [47]. The current analysis was controlled for complex survey design (clustering, weighting, and stratification) by using svyset command in STATA.

The spatial auto-correlation of neighbourhood values around a certain spatial location is measured by univariate Moran’s I. It determines the amount to which the data is spatially non-stationary and clustered. The local correlation between an outcome variable and specific geographical features is investigated using bivariate Moran’s I. While both univariate and bivariate Moran’s I attempt to evaluate geographical data similarities and dissimilarities, they are proven to be less helpful when spatial clustering is unequal [48]. The method to calculate the Moran’s *I* statistic is as follows:

$$\mathrm{Univariate}\ \mathrm{Moran}'\mathrm{s}\ \mathrm{I}=\frac{n}{S_O}\times \frac{\Sigma_i{\Sigma}_j{W}_{ij}\left({x}_i-\overline{X}\right)\left({x}_j-\overline{X}\right)}{\Sigma_i{\left({x}_i-\overline{X}\right)}^2}$$

Where x is the variable of interest and $\overline{X}$ is its mean of x; n is the number of spatial units; *W*_*ij*_ is the consistent weight matrix between observation i and j with zeroes on the diagonal; and *S*_*O*_ is the aggregate of all spatial weights, i.e. *S*_*O*_ = Σ_*i*_Σ_*j*_*W*_*ij*_

$$\mathrm{Bivariate}\ \mathrm{Moran}'\mathrm{s}\ \mathrm{I}=\frac{n}{S_O}\times \frac{\Sigma_i{\Sigma}_j{W}_{ij}\left({x}_i-\overline{X}\right)\left({Y}_j-\overline{Y}\right)}{\Sigma_i{\left({y}_i-\overline{Y}\right)}^2}$$

Where x and y are the variables of interest; $\overline{X}$ is the mean of x; $\overline{Y}$ is the mean of y; n is the number of spatial units; *W*_*ij*_ is the homogenous weight matrix between observation i and j with zeroes on the diagonal; and *S*_*O*_ is the aggregate of all spatial weights, i.e., *S*_*O*_ = Σ_*i*_Σ_*j*_*W*_*ij*_.

Moran’s-I values vary from 1 (perfect dispersion) to + 1 (perfect dispersion) (perfect correlation). A value of zero denotes a random spatial pattern. A negative (positive) spatial autocorrelation is shown by negative (positive) values. Points with similar attribute values are close together when spatial autocorrelation is positive, whereas closely related points are more dissimilar when spatial autocorrelation is negative [48].

The spatial-correlation of neighborhood values surrounding a certain spatial location is calculated using univariate LISA [49]. It indicates the extent to which the data has geographic randomization and clustering. The measure (*I*_*i*_) is given by the following:

$$\mathrm{Univariate}\ \mathrm{LISA}:{I}_i=\frac{n.\left({x}_i-\overline{X}\right)}{\Sigma_i{\left({x}_i-\overline{X}\right)}^2}{\Sigma}_j{w}_{ij}\left({x}_j-\overline{X}\right)$$

To investigate the relationship between specific characteristics of regions and the result variable, bivariate Local Indicator of Spatial Association (LISA) measures were calculated. The formula for bivariate LISA is presented as:

$$\mathrm{Bivariate}\ \mathrm{LISA}:{I}_i=\frac{n.\left({x}_i-\overline{X}\right)}{\Sigma_i{\left({y}_i-\overline{Y}\right)}^2}{\Sigma}_j{w}_{ij}\left({y}_i-\overline{Y}\right)$$

Based on the four quadrants of Moran’s scatter plots, four different forms of spatial auto-correlation were created:

- Hot Spots: districts with high values, with alike neighbors (High-High).
- Cold Spots: districts with low values, with analogous neighbors (Low-Low).
- Spatial Outliers: districts with high values, but with low-value neighbors (High-Low) and
- Districts with low values, but with greater values of neighbors (Low-High).

The spatial weights W_ij_ are non-zero when i and j are neighbors; else it residues zero [50]. The weights employed in the study are Queen Contiguity weights, which indicate whether or not spatial units share a boundary [50]. If the set of boundary points of element I is represented by the band (i), then the Queen Contiguity Weight is defined by:

$$\mathrm{Wij}=\left\{\begin{array}{c}1, bnd(i)\cap bnd\ (j)\ne \varnothing \\ {}0, bnd(i)\cap bnd\ (j)\ne \varnothing \end{array}\right.$$

This, however, raises the possibility of spatial units sharing only one boundary point (such as a shared corner point on a grid of spatial units) [51–53]. As a result, requiring that some positive amount of their border be shared is a stronger condition [51–53].

A collection of regression models was utilized to evaluate the significant determinants of aided skilled deliveries in India. The degree of autocorrelation in the error term was determined using the spatial ordinary least square (OLS) regression model [54]. We estimated the spatial lag model (SLM) and spatial error model (SEM) after the OLS validated spatial autocorrelation in its error term for the dependent variable (SEM) [54]*.* The fundamental premise of a geographical lag model is that observations of the result variable are influenced in nearby areas, whereas the spatial error model is used to assess the effect of factors that were not involved in the regression model but had an effect on the outcome variable [54]. The main change between the two models is that, unlike the spatial error model, the spatial lag model excludes the error term’s spatial dependence [55].

The simple equation for OLS is as follows:

$$\mathrm{Y}=\upalpha +\mathrm{BX}+\upvarepsilon$$

Where Y is the main outcome variable, X is the vector of predictor variables, and α is the model intercept, and β is the corresponding coefficient vector.

According to the spatial lag model, the units are geographically reliant on each other and lag behind each other in close spatial locations [54]. A typical spatial lag model can be inscribed as follows:

$${Y}_i=\delta \sum_{j\ne 1}{W}_{ij}{Y}_j+\beta {X}_j+{\varepsilon}_j$$

Here *Y*_*i*_ signifies the assisted skilled deliveries for the *i*^*th*^ district, δ is the spatial autoregressive coefficient, *W*_*ij*_ represents the spatial weight of proximity between district i and j, *Y*_*j*_ are the assisted skilled deliveries in the *j*^*th*^ district, *β*_*j*_ indicates the coefficient, *X*_*j*_ is the explanatory variable and εj is the residual [51–53].

On the other hand, the spatial error model examines the contribution of omitted variables that are not included in the model but might have a major impact on the study [54]*.* A Spatial Error Model (SEM) is stated as follows:

$${Y}_i=\beta {X}_j+\lambda \sum_{j\ne 1}{W}_{ij}{Y}_j{\varepsilon}_j+{\varepsilon}_i$$

Here, *Y*_*i*_ signifies the assisted skilled deliveries for the *i*^*th*^ district, λ is the spatial autoregressive coefficient, *W*_*ij*_ represents the spatial weight of proximity between district i and j, *Y*_*j*_ is assisted skilled deliveries in the *j*^*th*^ district, *β*_*j*_ symbolizes the coefficient, *X*_*j*_ is the predictor variable and *ε*_*i*_ is the residual [51–53]. QGIS (Version 3.22.1) was used to merge the datasets and to create the prevalence and hotspot map. Moreover, GeoDA was used to conduction spatial analysis. Both the software’s are open source in nature.

## Results

Table 1 presents univariate and bivariate Moran’s I value of the outcome and explanatory variables for women in India. The univariate Moran’s I value for SBA among women was 0.54, which represents a high spatial auto-correlation of deliveries conducted by SBA over 640 districts of India. The highest univariate Moran’s I value for the explanatory variable was for women from poor socio-economic profiles (0.74), followed by four or more ANC (0.73) and women from the Hindu religion (0.71). Additionally, the highest bivariate Moran’s I (SBA vs explanatory variable) value was for SBA vs four or more ANC (0.49) followed by SBA vs women from poor socio-economic status (− 0.43) and SBA vs first birth order (0.43).

**Table 1:** Univariate and Bivariate Moran’s I Values for the outcome and explanatory variables for women in India, (*N* = 640)

Variables	Univariate	Bivariate (Delivery conducted by SBA)
	Moran's I (p-value)	Moran's I (p-value)
Delivery conducted by SBA (%)	0.54 (0.001)	–
Educated (%)	0.67 (0.001)	0.33 (0.001)
Mass media exposure (%)	0.66 (0.001)	0.42 (0.001)
Four or more ANC (%)	0.73 (0.001)	0.49 (0.001)
First birth order (%)	0.61 (0.001)	0.43 (0.001)
Female headed HH (%)	0.58 (0.001)	−0.01 (0.258)
SC/ST (%)	0.57 (0.001)	−0.23 (0.001)
Non-Hindu (%)	0.71 (0.001)	−0.21 (0.001)
Poor (%)	0.74 (0.001)	−0.43 (0.001)
Rural (%)	0.42 (0.001)	−0.25 (0.001)

%: percentage, *SBA:* skilled birth attendant, *ANC:* antenatal care, *HH:* household, *SC/ST:* scheduled caste/scheduled tribe

Table 2 depicts the estimates from the spatial regression model for estimating the spatial association between deliveries conducted by SBA by background factors among women in India. OLS estimates are the adjusted ones, but these estimates are not controlled for spatial correlations. However, Spatial Lag Model (SLM) and Spatial Error Model (SEM) provide estimates adjusted spatial correlations hence termed as spatial auto-regressive models. At first, it is important to verify the best fit model using AIC and adjusted R square values. It was found that the best fit model was the spatial error model with the lowest AIC value (4483.9) and the highest R square value (0.81).

**Table 2:** Spatial regression model for estimating the spatial association between delivery conducted by SBA by background factors among women in India, (N = 640)

Predictors	Delivery conducted by SBA		
	OLS (Coef. (p-value))	SLM (Coef. (p-value))	SEM (Coef. (p-value))
Educated	− 0.089 (0.015)	−0.079 (0.028)	0.062 (0.136)
Mass media exposure	0.239 (0.000)	0.234 (0.000)	0.252 (0.000)
Four or more ANC	0.217 (0.000)	0.197 (0.000)	0.252 (0.000)
First birth order	0.520 (0.000)	0.489 (0.000)	0.611 (0.000)
Female headed HH	0.130 (0.022)	0.171 (0.002)	0.137 (0.029)
SC/ST	−0.092 (0.000)	−0.084 (0.000)	−0.034 (0.111)
Non-Hindu	−0.100 (0.000)	−0.082 (0.000)	− 0.052 (0.008)
Poor	−0.016 (0.591)	0.005 (0.864)	0.045 (0.185)
Rural	0.015 (0.496)	0.006 (0.784)	0.044 (0.044)
**N**	640	640	640
**ρ**		0.140 (0.000)	
**Lamda**			0.739 (0.000)
**AIC**	4713.6	4691.1	4483.9
**Adjusted R square**	0.68	0.69	0.81

*AIC:* Akaike information criterion, *OLS:* Ordinary least-square, *SLM:* Spatial lag model, *SEM:* Spatial error model, *SBA:* skilled birth attendant, *ANC:* antenatal care, *HH:* household, *SC/ST:* Scheduled Caste/Scheduled Tribe; *Coef*: Coefficient

The spatial error model revealed that with a 10% increase in the mass media exposure in a particular district, is associated with a 2.5% increase in deliveries conducted by a SBA. Interestingly, also with the 10% increase in the four or more ANC in a particular district, is associated with a 2.5% increase in deliveries conducted by a SBA. Surprisingly, if there was a 10% increase of women with first birth order in a particular district, the association gets increased by 6.1% when deliveries are conducted by SBA. Suppose the district experienced an increase of 10% household as female-headed as the deliveries conducted by SBA would significantly increase the association by 1.4%. An increase of 10% of non-Hindu households in a district is associated with a 0.5% decline in deliveries conducted by a SBA. Lastly, an increase of 10% of the rural population in a district is associated with a 0.4% increase in deliveries conducted by a SBA.

**Fig. 1.**
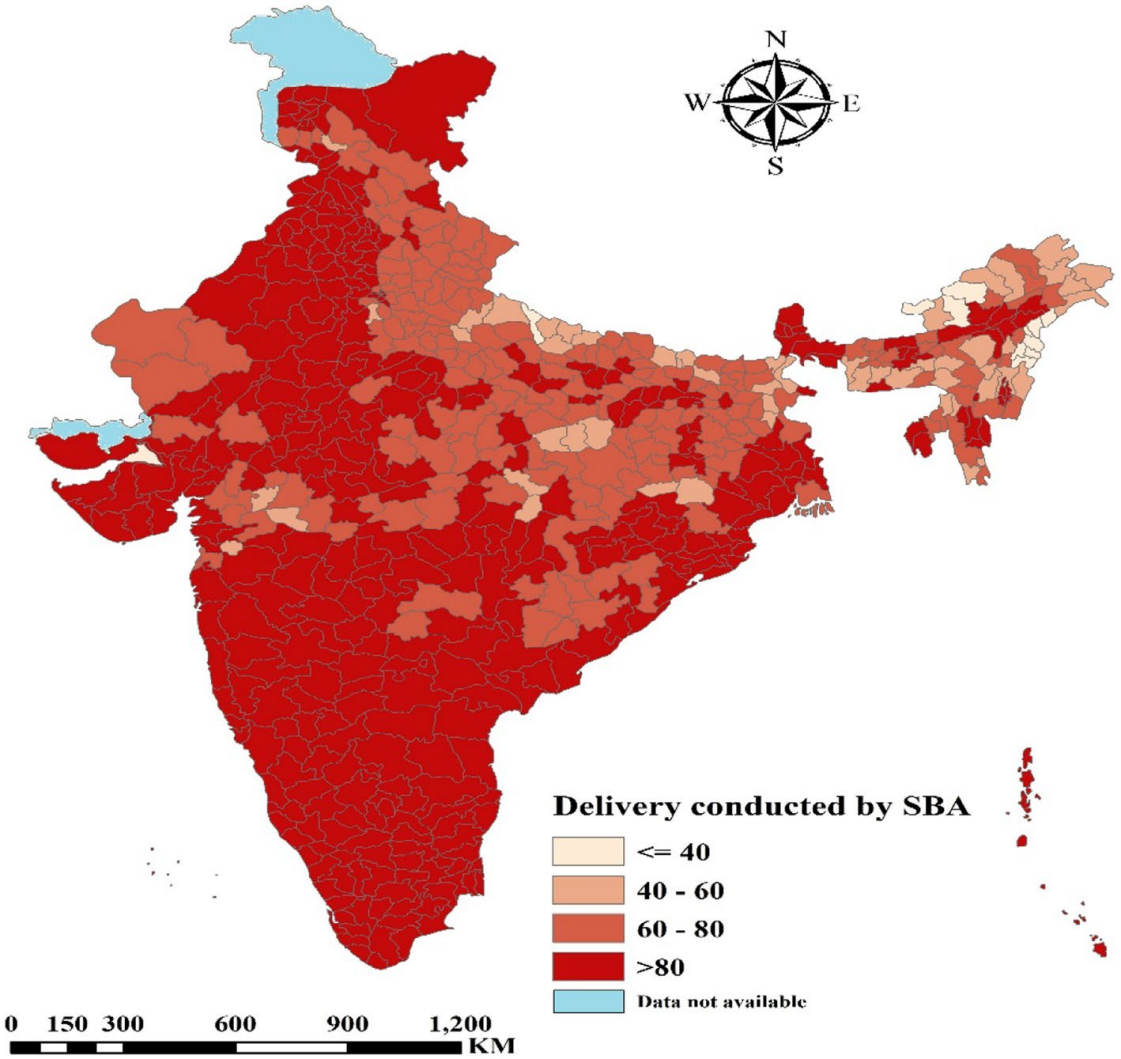
Percentage distribution of deliveries conducted by SBA among women in districts of India. (The map was created by the author’s using QGIS (Version 3.22.1), QGIS is an open source software hence license was not required)

Figure 1 presents the percentage distribution of deliveries conducted by SBA among women in districts of India. It was found that women from the larger part of India were delivered by SBA. Some parts of Uttar Pradesh, Madhya Pradesh, Jharkhand, West Bengal and certain regions of North Eastern India had less than 40% of deliveries conducted by SBA.

**Fig. 2.**
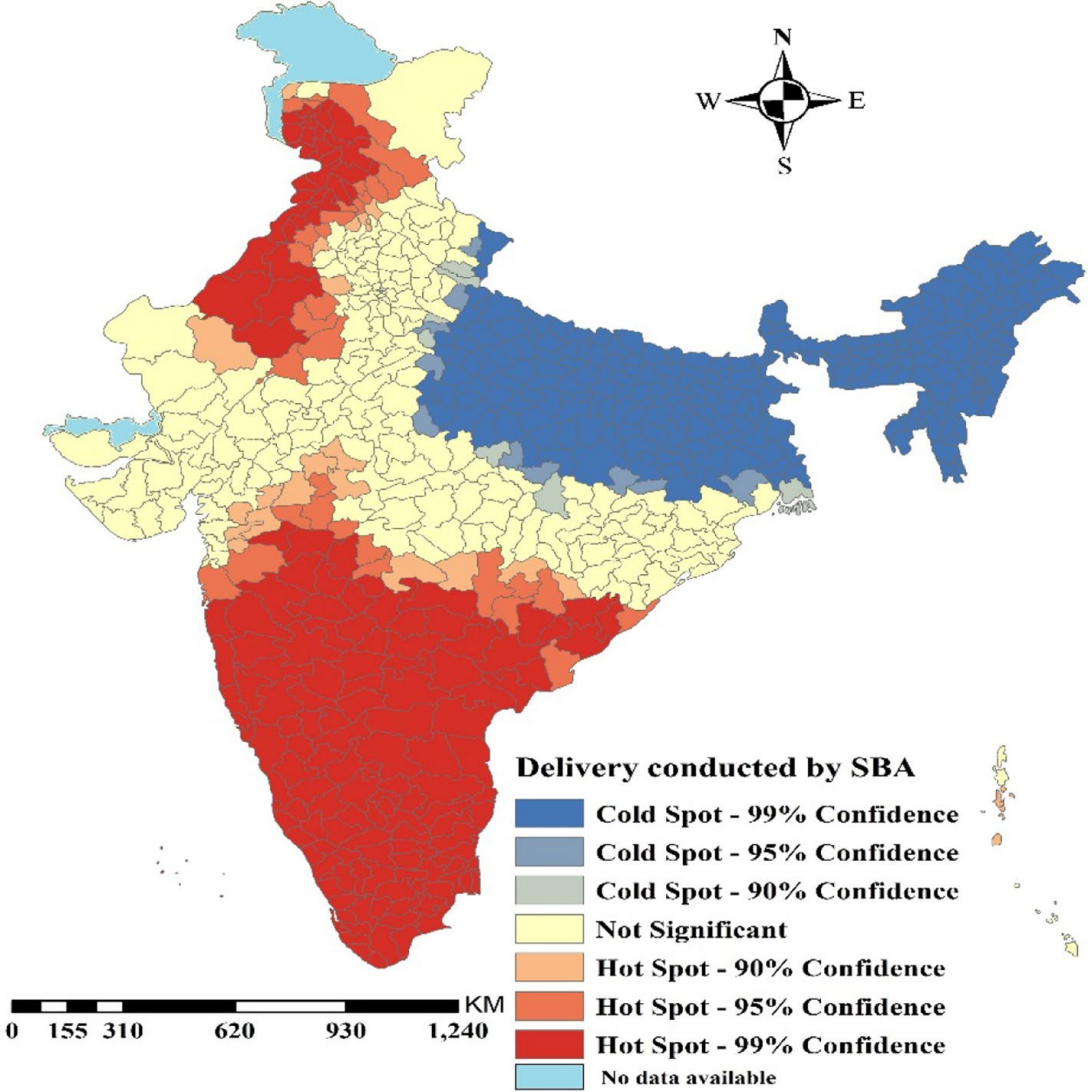
Hotspot map for deliveries conducted by SBA among women in districts of India. (The map was created by the author’s using GeoDA, GeoDA is an open source software hence license was not required)

Figure 2 presents the hotspot analysis for deliveries conducted by SBA among women in India. It was clearly visible in the respective map that delivery conducted by SBA was highly concentrated in the Southern part of India including, parts of Orissa, Madhya Pradesh, Rajasthan, Haryana, Punjab and Jammu & Kashmir. However, Uttar Pradesh, Bihar, West Bengal and the whole north-eastern part of India had formed a cold spot that depicted a low percentage of deliveries conducted by SBA.

**Fig. 3.**
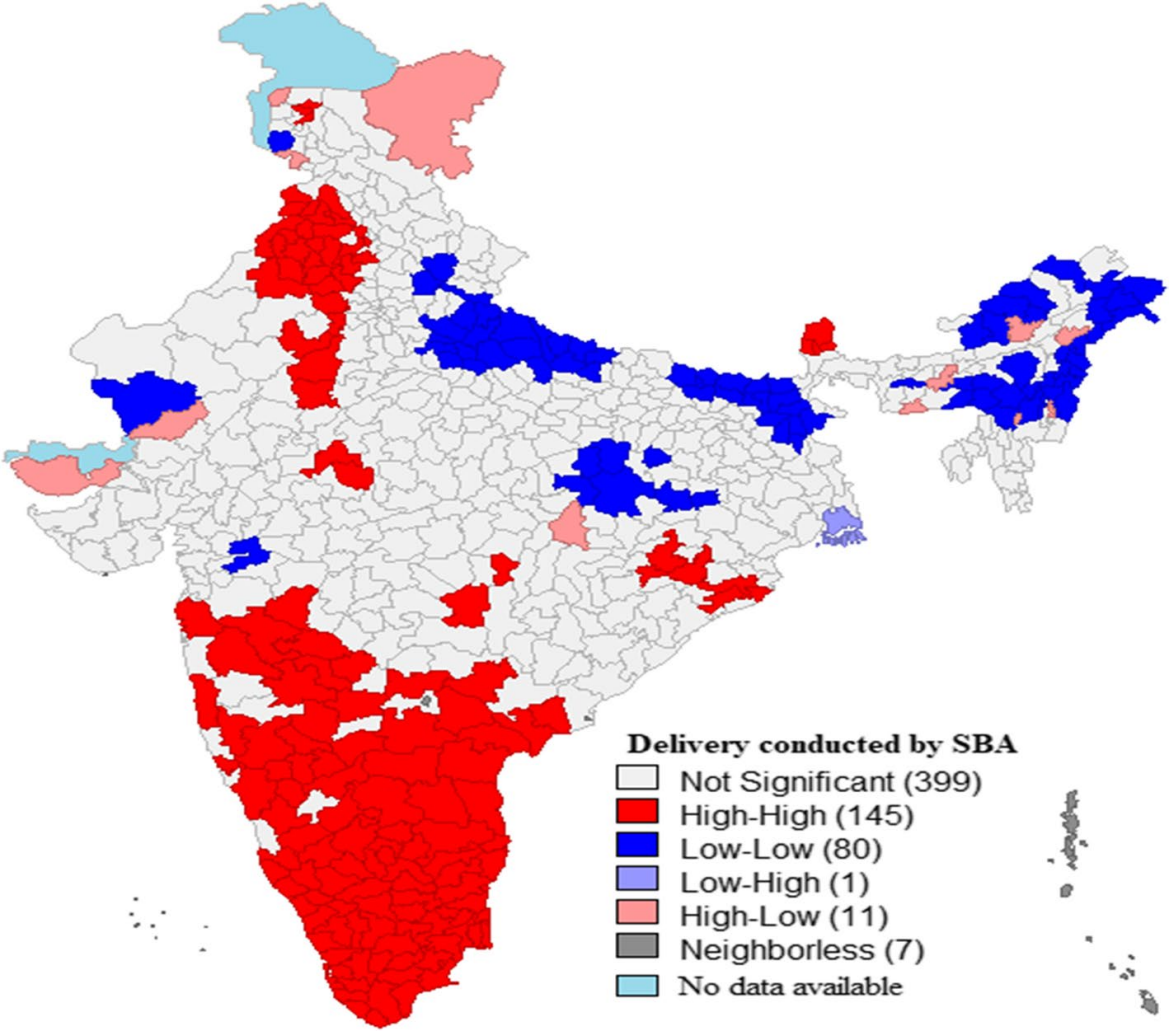
Univariate LISA map for deliveries conducted by SBA among women in India, (N = 640). (The map was created by the author’s using GeoDA, GeoDA is an open source software hence license was not required)

Figure 3 represents the univariate LISA maps for background characteristics for women aged 15-49 years in India. There were 145 hotspots (one hotspot represents one district) for deliveries conducted by SBA among women in India, which included almost the entire southern part of India that comprised of Maharashtra, Andhra Pradesh, Karnataka, Kerala and Tamil Nadu, including certain districts of Orissa, Sikkim, Madhya Pradesh, Rajasthan, Haryana, Punjab and Jammu & Kashmir. Univariate LISA maps for background characteristics for women in India in presented in figure-S1 (supplementary file).

**Fig. 4.**
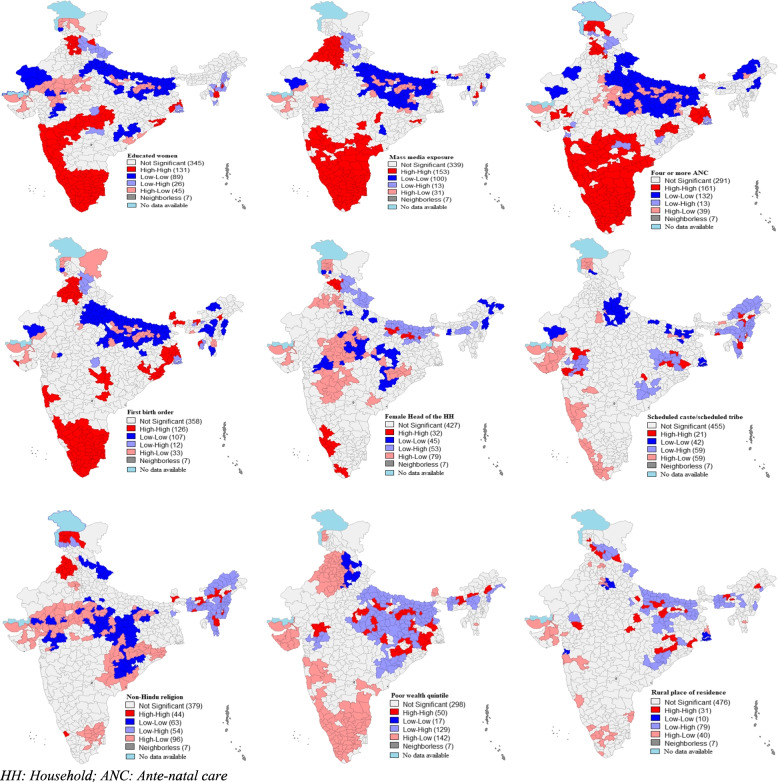
Bivariate LISA maps for deliveries conducted by SBA by background characteristics, India (N = 640). (The map was created by the author’s using GeoDA, GeoDA is an open source software hence license was not required)

Figure 4 represents the bivariate LISA maps for delivery conducted by SBA by background characteristics among women in India. About 131 out of 640 districts formed hotspots for deliveries conducted by SBA and educated women, which include Kerala, Tamil Nadu, Goa, Karnataka, Maharashtra, Madhya Pradesh and certain parts of Himachal Pradesh, Orissa, West Bengal, Mizoram and Manipur. Nearly 153 out of 640 districts formed hotspots for deliveries conducted by SBA and media exposure which consist of Himachal Pradesh, Haryana, Punjab, Sikkim, Mizoram, Manipur, Orissa, Madhya Pradesh, Telangana, Andhra Pradesh, Karnataka, Maharashtra, Goa, Kerala and Tamil Nadu. About 161 out of 640 districts formed hotspots for deliveries conducted by SBA and four or more ANC, which includes Kerala, Tamil Nadu, Karnataka, Goad, Maharashtra, Andhra Pradesh, Telangana, Gujarat, Madhya Pradesh, Orissa, West Bengal, Sikkim and parts of Jammu & Kashmir, Himachal Pradesh and Punjab. Nearly 126 out of 640 districts were forming hotspots for first birth order and deliveries conducted by SBA, which includes Kerala, Tamil Nadu, Karnataka, Maharashtra, Orissa, West Bengal, Madhya Pradesh, Sikkim, Assam, Himachal Pradesh, Haryana and Punjab. Out of 640 districts, 32 were forming the hotspot for females as household heads and deliveries conducted by SBA, which includes Kerala, Karnataka, Himachal Pradesh and some parts of Bihar. About 21 out of 640 districts were forming the hotspot for women from SC/ST caste and deliveries conducted by SBA, which includes some districts from Maharashtra and Gujarat, which is western India region, some districts from Orrisa, Chhattisgarh, and Jharkhand that is from the eastern part of India and North-eastern regions. Nearly 44 out of 640 districts were forming the hotspot for women from non-Hindu religion and deliveries conducted by SBA, which includes the states from Jammu & Kashmir, Punjab, and north-eastern states. Nearly 50 out of 640 districts were forming the hotspot for women from poor socio-economic status and deliveries conducted by SBA, which includes from central and eastern states like Chhattisgarh, Jharkhand, Orrisa, Madhya Pradesh, Uttar Pradesh, Bihar and some districts from Meghalaya and Assam. About 31 out of 640 districts were forming the hotspot for women from a rural place of residence and deliveries conducted by SBA, which includes the states such as Himachal Pradesh, Uttarakhand, Bihar, Jharkhand and Chhattisgarh and Madhya Pradesh.

## Discussion

Worldwide various studies using spatial distribution models have helped in identifying geographic clusters of good and poor maternal health outcomes [56–58]. Like a study in Indonesia found that a woman’s area of residence, i.e., rural or urban determined whether she received antenatal care or not [59]. In Mexico, the highest level of maternal mortality was found in the most socially vulnerable municipalities [60]. In India, studies have used geo-spatial analysis to investigate the problems related to the reproductive health of women at the district level [44, 61, 62]. The merit of using geo-spatial techniques lies in the fact that policies can be planned at the micro-level. Furthermore, literary evidences indicate that application of spatial techniques provide policymakers with the capability to identify high priority areas that require more maternal health services which helps in improvement of maternal health [63, 64] along with producing fine spatial scale estimates of reproductive and maternal indicators in low- and middle-income countries [65]. Since India exhibits a wide regional variation of maternal mortality ratio and limited use of SBAs for deliveries, therefore, the present study attempts to understand the spatial distribution of deliveries by SBAs across 640 districts in India as well as to identify the background characteristics that affect its utilization. By analyzing the kid’s file of the fourth round of the National Family Health Survey, the key results of the study are discussed below.

First, the results of the spatial error model helped to identify the background characteristics that might improve the utilization of SBA in India. For instance, mass media exposure, four or more than four ANC visits, households with female heads significantly increase the deliveries by an SBA. A community-based cross-sectional study in Tamil Nadu, India, reveals that women with exposure to mass media are more likely to go for institutional deliveries [66]. Another study suggests that birth preparedness like saving up of money, identifying SBA and transportation is more among women who are exposed to mass media [67]. Another crucial factor for deliveries conducted by an SBA is regular ANC visits during pregnancy. The study finding is compatible with other empirical evidence that shows women who attend ANC services are assisted by an SBA during deliveries [68–70]. Similar to other studies, this study also found that households with female heads are also the households to have an increase in deliveries by SBAs [71, 72]. Possible reasons could be (i) if the pregnant woman is herself the head of the household, then she has control over the household resources (like keeping money aside) that has a significant effect on healthcare usage [73] and (ii) if either her mother or mother-in-law is the household head then apart from the fact that senior women in the household are decision-makers in issues related to pregnancy, the pregnant woman can easily communicate about pregnancy-related communications [74]. In both cases, it leads to utilizing an SBA for birth delivery.

Second, a distinct regional variation in deliveries by SBAs is observed through the maps. The high percentage of deliveries by SBAs are observed in Southern India, parts of Orissa, Madhya Pradesh, Rajasthan, Haryana, Punjab and Jammu & Kashmir, whereas Uttar Pradesh, Bihar, West Bengal, and North-Eastern India form a cold spot. However, when we look into the results of bivariate LISA maps for delivery conducted by SBAs by background characteristics of women in India, we find that even among the cold spots, states like Uttar Pradesh and Bihar do not perform well. For instance, when we consider background characteristics like women’s education, ANC visits, first birth order, West Bengal (being a part of the cold spot states) is far better than Uttar Pradesh & Bihar. It’s a matter of grave concern that none of the background characteristics, especially first birth order, influences deliveries by SBAs in these two states. This is in contrast to findings from studies around the globe that have shown the remarkable influence of birth order in the utilization of an SBA for delivery (23, 36, 37). Again, among the North-eastern states of India, deliveries by SBAs are low if the states have high rural households, and no females as household heads.

Literature focusing on low utilization of SBA for deliveries mentions several barriers like financial, social, regional and cultural [12]. Other factors range from the scarcity of skilled providers, poor health-system infrastructure, substandard quality of care to women’s reluctance to use maternity care where high cost is involved [6]. Further, lack of transportation, distance from Maternal and Child Health (MCH) centers, unavailability of doctors at the MCH centers are considered to be major obstacles to reach health facilities in hilly areas [75, 76].

### Strength and limitation of the study

Given the wide regional and socio-economic inequality in availing maternity care services in India, the present study provides estimates of delivery conducted by SBA’s at the district level. The regional spatial approach of the study will help policymakers in devising policies that will ensure district-specific interventions regarding maternity health care services. Moreover, by using a recently published nationally representative sample of a well-known large-scale survey in India, the study results can be generalized well. The study has its limitation too. First, because of the cross-sectional nature of the data, we could not draw any causal relationship between the variables. Secondly, we cannot rule out the possibility of response bias because the respondents’ attitudes and manners could have affected their responses. Thirdly, since the study is based on secondary data, we could not explore community-level factors like availability of physical infrastructures at the facility levels, poor attitude and communication between health workers and patients that might influence the health care seeking behavior of the women. In future, a more in-depth qualitative study can explore these factors.

### Policy implication

As already discussed, India is a country that displays wide regional variation in the use of SBAs. Therefore, it makes sense to focus on district-specific interventions that will strengthen the health facilities and infrastructure for that particular district, thereby minimizing the existing regional variation. This will also enhance the quality of care and access to maternal health care services. We also acknowledge that for a better recommendation, evidence has to come from a meta-analysis. Therefore, in future, the present paper can be counted as input for meta-analysis.

## Conclusion

The present study highlights the important role of ANC visits, mass media, education, and female household headship that augments the use of an SBA for delivery. Policymakers, while devising specific interventions to access maternal health care services, can include these factors. Attention should be given to promoting regular ANC visits and strengthening women’s education. The results also emphasize that the provision and utilization of maternal health care services are constrained by a woman’s geographic location, especially in the hilly North-Eastern states of the country. Thus, a more comprehensive approach involving socio-economic and geographic determinants is needed in India.

## Supplementary Information

**Additional file 1: Figure S1**. Univariate LISA maps for background characteristics for women in India (*N* = 640). (The map was created by the author’s using GeoDA, GeoDA is an open source software hence license was not required).

## Data Availability

The study utilizes a secondary source of data that is freely available in the public domain through https://dhsprogram.com/what-we-do/survey/survey-display-355.cfm.

## Abbreviations

SBA: Skilled Birth Attendant
LISA: Local indicators of spatial association
OLS: Ordinary Least Square SLM Spatial Lag Model
SEM: Spatial Error Model
AIC: Akaike information criterion
NFHS: National Family Health Survey
ANC: Antenatal care
HH: Household
SC/ST: Scheduled Caste/Scheduled Tribe
