# Supplementary material for: Spatial inequalities in skilled birth attendance in India: a spatial-regional model approach
Source: BMC Public Health. 2022 Jan 12;22:79. doi: 10.1186/s12889-021-12436-7 (PMC8756682; doi:10.1186/s12889-021-12436-7)
Supplement: Supplementary file 1 — Additional file 1: Figure S1. Univariate LISA maps for background characteristics for women in India (N = 640). (The map was created by the author’s using GeoDA, GeoDA is an open source software hence license was not required). [file 12889_2021_12436_MOESM1_ESM.docx]

| **Figure-S1.** Univariate LISA maps for background characteristics for women in India (N=640)  (The map was created by the author’s using GeoDA, GeoDA is an open source software hence license was not required) | | |
| --- | --- | --- |
| 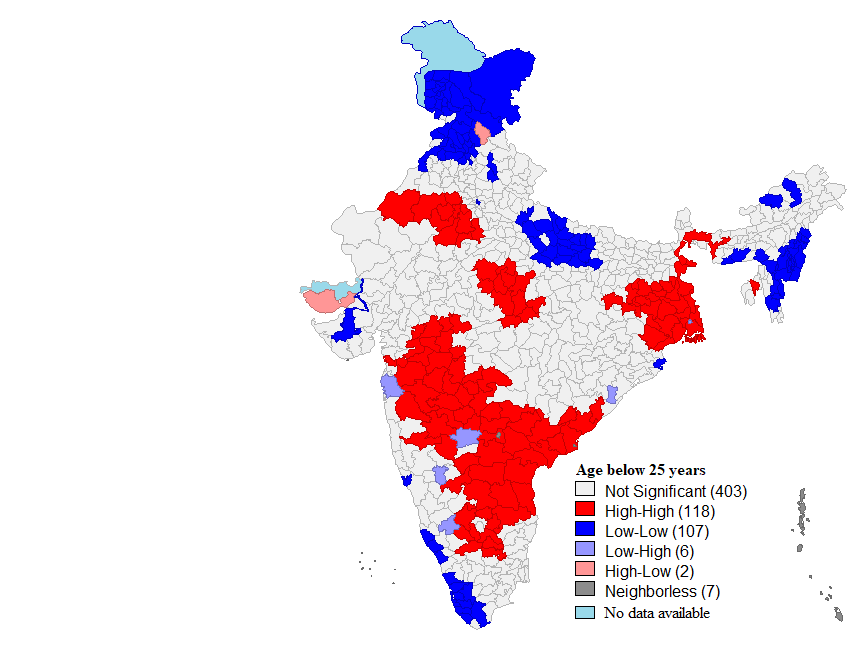 | 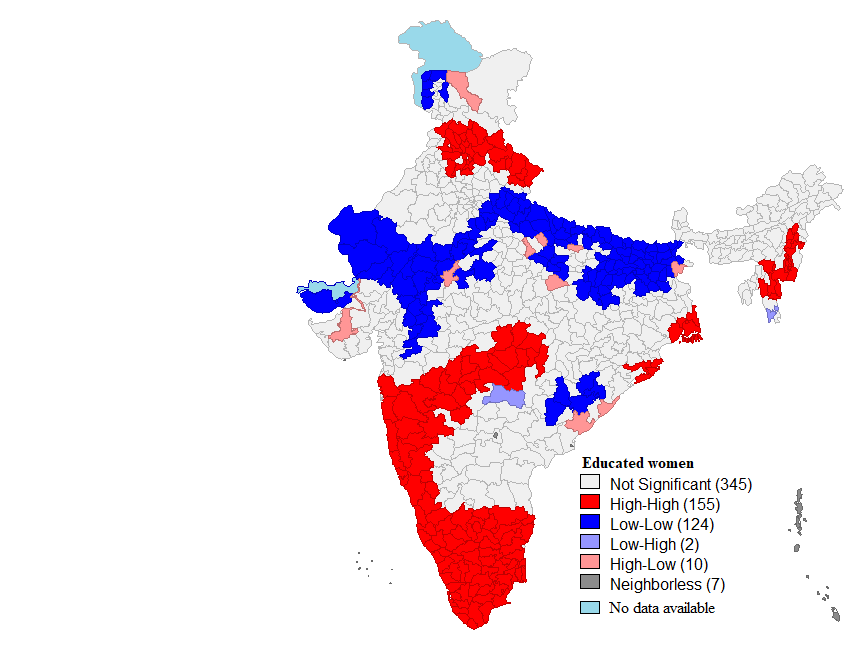 | 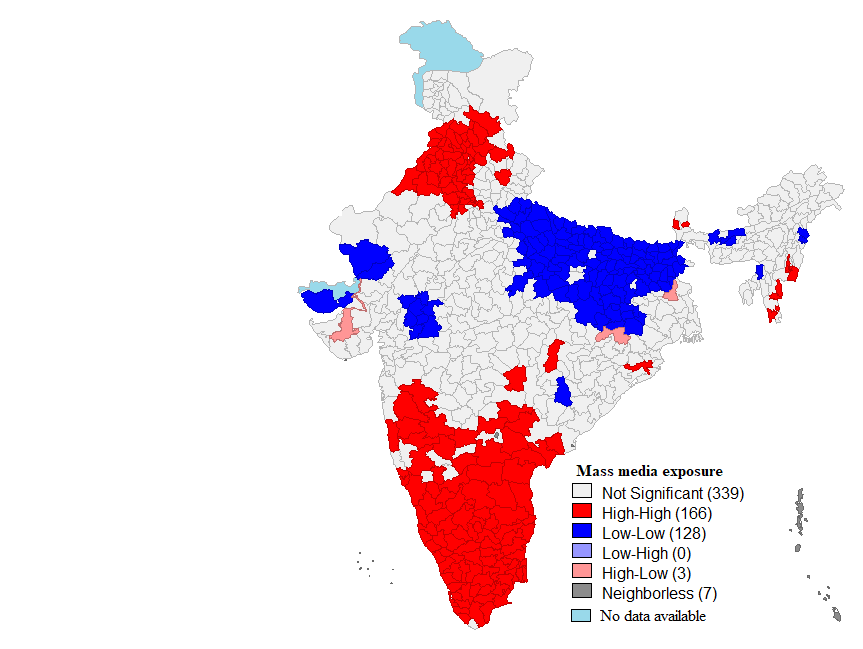 |
| 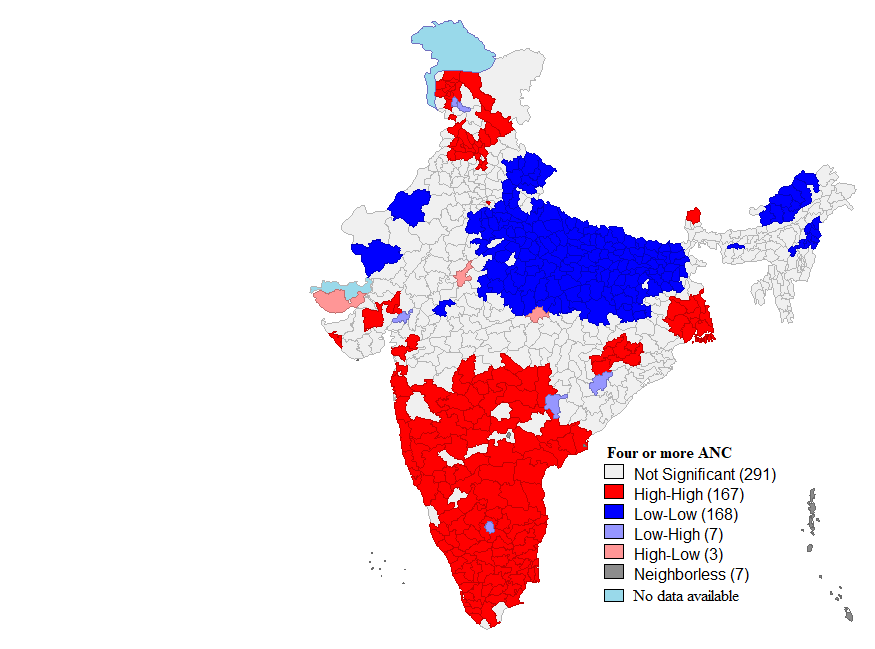 | 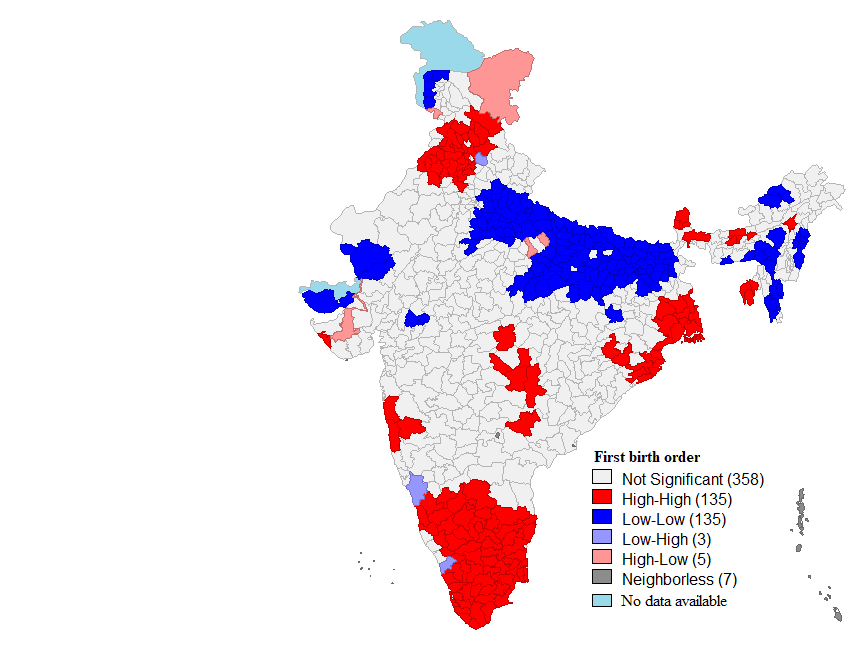 | 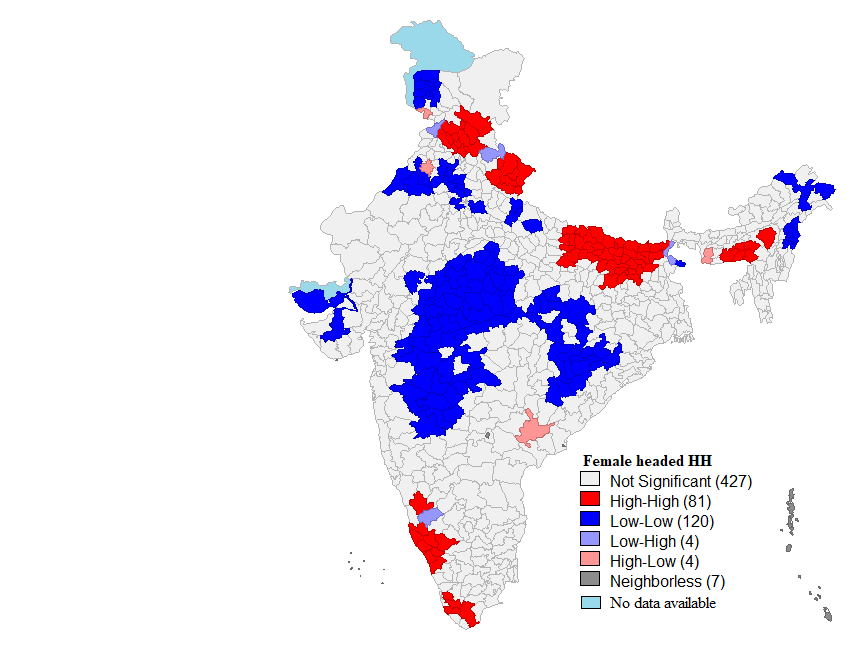 |
|  |  |  |
| 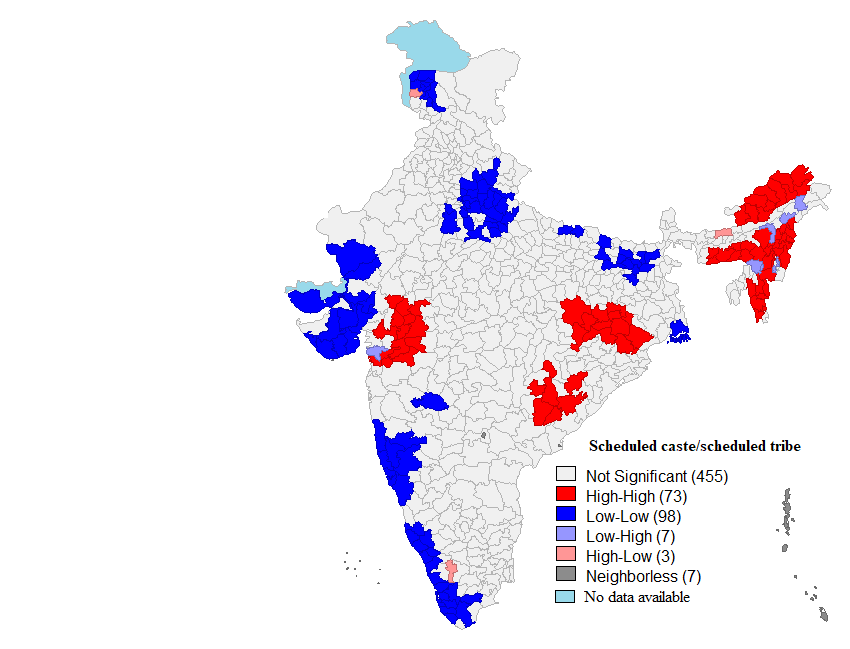 | 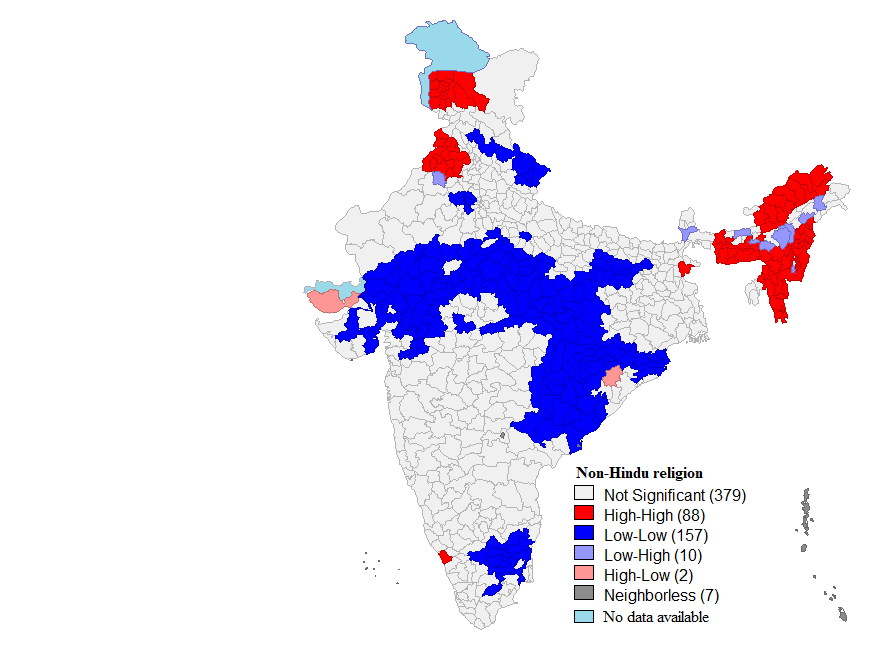 | 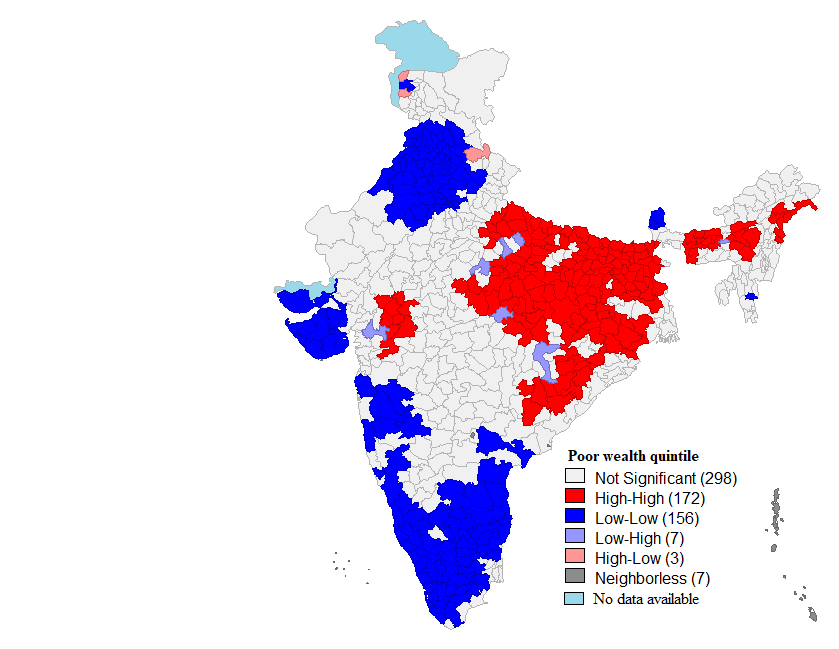 |
| 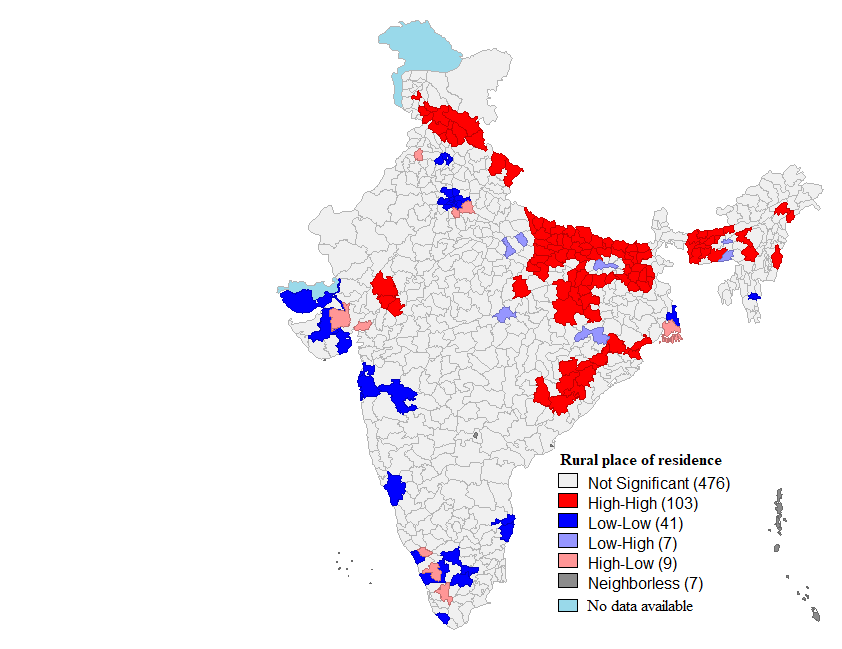 |  |  |

*HH: Household; ANC: Ante-natal care*

***Source:*** *Author’s contribution*
